# Reorienting women's health in low- and middle-income countries: the case of depression and Type 2 diabetes

**DOI:** 10.3402/gha.v7.22803

**Published:** 2014-01-09

**Authors:** Emily Mendenhall, Lesley Jo Weaver

**Affiliations:** 1 Science, Technology, and International Affairs Program, School of Foreign Service, Georgetown University, Washington, DC, USA; 2 Department of Anthropology, Emory University, Atlanta, Georgia, USA

**Keywords:** women's health, depression, Type 2 diabetes, life course, social determinants, epidemiological transition

## Abstract

Women's health in low- and middle-income countries (LMICs) has historically focused on sexual and reproductive health. However, understanding how women acquire, experience, and treat non-reproductive health conditions, such as non-communicable diseases, has become a fundamental public health concern. Special attention to the social determinants of LMIC women's health can provide socially and culturally relevant knowledge for implementation of policies and programs for women increasingly confronting these ‘New Challenge Diseases’. This article uses the example of depression and Type 2 diabetes comorbidity to illustrate how attending to the social determinants of mental and physical health beyond the reproductive years contributes to a more holistic agenda for women's health. For instance, we must address the plurality of experiences that shape women's health from social determinants of depression, such as gendered subjugation within the home and public sphere, to the structural determinants of obesity and diabetes, such as poor access to healthy foods and health care. Attending to the complexities of health and social well-being beyond the reproductive years helps the women's global health agenda capture the full spectrum of health concerns, particularly the chronic and non-communicable conditions that emerge as life expectancy increases.

The contemporary landscape of women's health in low- and middle-income countries (LMICs) is more complex than public health approaches in previous decades reflected, when the focus was primarily on sexual and reproductive health. As populations age, no longer are sexual and reproductive health the dominant themes that shape how women can live longer, healthier lives. Instead, a combined perspective of the social determinants of mental and physical health across the life course comes to the forefront. Understanding how women acquire, experience, and treat non-reproductive health conditions, such as non-communicable diseases, over the course of their lives is particularly important for women living in resource-constrained settings, who are socially and economically marginalized and often experience limited access to healthcare. This article uses the example of depression and Type 2 diabetes comorbidity to illustrate how attending to the social determinants of mental and physical health beyond the reproductive years contributes to a more holistic agenda for women's health.

This shift in priorities requires that we break down the traditional distinctions between ‘chronic’ and ‘acute’, ‘communicable’ and ‘non-communicable’ diseases because in fact they often occur together. For instance, diabetes and tuberculosis not only coexist within a given population but also can coexist within a single individual. Likewise, over- and under-nutrition can exist simultaneously in communities, households, or even individuals during different phases of their lives. In light of these complex scenarios, Knaul and Frenk have suggested that we rethink public health paradigms for the challenges of aging populations as ‘New Challenge Diseases’ rather than ‘non-communicable diseases’ (1). This approach requires that we move beyond diseased-focused silos in public health. Instead, we must address the plurality of experiences that shape women's health from social determinants of depression, such as gendered subjugation within the home and public sphere, to the structural determinants of obesity and diabetes, such as poor access to healthy foods and health care. Women living in LMICs require special attention not only because their experiences are unique to women living in affluent nations but also because such limited research is available on their social and health problems. Bias of research from high-income nations may construe LMIC women's experiences and contribute not only to knowledge displaced from women's social experiences but also policies and programs that do not reflect the social, economic, and cultural factors surrounding women's mental and physical health problems in LMICs.

As opposed to traditional disease-based approaches in medicine and public health, a life course approach encompasses the powerful role of social and economic determinants of health in women's lives from infancy to old age (2, 3). This approach is particularly important for women who may experience disproportionate social disadvantage, gendered discrimination, and chronic, untreated depression when compared to men (4). Indeed, new global data demonstrates that women's health is overall poorer than their male counterparts around the world (5), and this is largely due to socially driven inequalities. Recognizing this is crucial for understanding and managing chronic diseases, which typically have complex etiologies rooted in long-term lifestyle choices as well as intergenerationally heritable characteristics, both genetic and behavioral. A life course perspective acknowledges, for instance, that social and economic problems related to poverty both fuel poor health and result from it, creating cycles that are difficult to break.

We present complexities of the comorbidity between Type 2 diabetes and depression to illustrate the need for a life course perspective in women's health. Type 2 diabetes, an adult-onset chronic disease, is widely known as a disease of ‘modernization’ that is emerging in LMICs and shifting from affluent to lower income groups all over the world (6, 7). Biologists and epidemiologists identify depression as both a cause and consequence of diabetes (8, 9), while medical social scientists have elucidated some of the complex socioeconomic and psychophysiological pathways linking the two chronic conditions (10). Despite increasing diabetes prevalence in LMICs, the research on social experiences of those living with diabetes, depression, and their comorbidity is limited. The few existing qualitative studies suggest that experiences differ between men and women (11) as well as between income groups (12).

Social and economic determinants of women's health are fundamental in the relationship of depression and diabetes, particularly among people of lower socioeconomic status (6, 13). As underscored in the 2010 Global Burden of Disease studies, experiences of social problems such as various forms of interpersonal abuse, and psychological problems such as depression and anxiety, have escalated either by detection or actual incidence among women on a global scale (5). Stress throughout the life course rooted in childhood trauma, abuse, or the chronicity of poverty may be key risk factors for depression and/or poor eating and activity patterns that lead to obesity and its complications, such as Type 2 diabetes (10). Complicating matters is the dual burden associated with living in poverty in rapidly modernizing cities that make unhealthy foods accessible and affordable, fueling obesity epidemics in LMIC settings (7). These inequalities create a negative feedback loop, whereby social and economic problems increase the likelihood of developing depression, diabetes, and their overlap, and these illnesses together promote the development of diabetes-related complications such as loss of limbs or eyesight and subsequent physical disability, further compounding socioeconomic inequalities (10). Finally, because of stigma and limited mental healthcare services in LMICs (14), women experiencing this comorbidity are more likely to seek care for diabetes than for depression, leaving half of the comorbidity unaddressed (11).

In India, home to the second largest population of people with Type 2 diabetes in the world (13), recent epidemiological and qualitative data suggest that the illness is becoming more prevalent among the middle classes and working poor (15). In tandem, mental healthcare is limited (16). Despite active research and policies aimed at addressing chronic and mental health diseases in India (17), there remains a large gap in knowledge about how these conditions afflict various Indian communities in their everyday lives, especially poor women. Qualitative research on depression and diabetes in India indicates that lower income people experience higher rates of social stress and depression, and poorer access to health care (12). Such research also underscores the powerful role that gendered social roles play in shaping women's mental health and diabetes outcomes (18). For example, gendered behavioral norms orient Indian women strongly toward the care of others, and therefore away from the self-care activities that are usually integral to diabetes management (11). Maintaining these other-care-oriented roles appears to be good for diabetic women's mental, but not physical, health.

The recognition of social forces as part of diabetes and depression etiology in India and other LMICs presents new challenges for public health because it underscores that medicating these complex illnesses does not fully address them. Finding a better public health solution to comorbidities like Type 2 diabetes and depression will likely only occur when we understand the limitations, and harness the power of, cultural beliefs and social conditions to shape behaviors that affect chronic diseases: how people eat, move, and medicate; how economic conditions may function as a barrier to treatment; and how depression may complicate a chronic disease, both socially and physically (7).

The comorbidity between depression and diabetes among women in LMICs is but one example of the ways in which women's non-reproductive health concerns deserve more prominence in global health. It also presents a strong case for increased attention to social and psychological determinants of women's health over the life course. The present lack of such perspectives in women's global health may result from limited funding for non-reproductive issues, lack of interest, or may simply be another manifestation of the great information gap between high-income countries and LMICs. Regardless, it should be a priority of future research, programming, and policy.

## Focusing on health, not disease

Why should diabetes and depression comorbidity be on the women's health agenda? Depression has only become a major global health concern in the past decade, and has proven very difficult to address, not least because of stigma and limited human resources for mental healthcare. This is especially true for women in LMICs, whose access to mental healthcare may be virtually non-existent, and whose care-seeking behaviors and budgets typically include little, if any, room for mental healthcare. Moreover, most LMICs’ health systems are poorly equipped to meet the complex prevention and management challenges associated with chronic conditions like diabetes and mental illnesses because, until very recently, infectious diseases were the dominant population health concerns.

The Movement for Global Mental Health's often-cited slogan, that there is ‘no health without mental health’ (14), emphasizes the need for integrated mental and physical healthcare systems to combat the next generation of public health problems. This would require an ideological and organizational shift in biomedicine, which has until recently viewed physical and mental health as separate categories of pathology requiring separate treatments, but would likely open up new avenues for cost-effective treatment. The WHO mental health Gap Action Program (mhGAP), for instance, suggests steps by which mental illness diagnosis and treatment can be integrated into primary care settings, and many initiatives are working to actualize this goal in LMICs (e.g. PRIME: http://www.prime.uct.ac.za/). With relatively little additional investment, basic mental healthcare could also be integrated into existing diabetes care guidelines. Such an approach is particularly important for women who face a higher burden of social problems and mental illness, which influence diabetes self-care and health outcomes. Yet, until a more integrative approach is adopted within clinics and public health agendas, healthcare silos will dominate global health dialogues, funding structures, and disease-focused (as opposed to *health*-focused) campaigns.

As the co-occurrence of mental and physical health problems gains recognition in the public health agenda, a more nuanced understanding of sociocultural influences on women's lifetime health is crucial. This is particularly important in LMIC settings where women face not only great social disadvantage but also an increasing burden of mental and physical health problems. A life course perspective requires acknowledging that women's mental and physical health are closely linked with cultural beliefs, social experiences (both past and present), and economic conditions over time. It also recognizes that women's health status shapes their social and economic conditions, for better or worse. Strategic points of intervention can improve women's social and emotional well-being across decades, which could then empower them to identify and care for their own health problems more effectively. In this way, integrating a social and psychological approach into health agendas, from the clinical to the policy level, can make a big impact.

**Main findings**

- Moving beyond disease-focused silos in public health requires that we attend to the plurality of experiences that shape women’s health from social determinants of depression, such as gendered subjugation within the home and public sphere, to the structural determinants of obesity and diabetes, such as poor access to health foods and health care.
- Complexities demonstrated by the comorbidity of depression and type 2 diabetes illustrate the need for a life course perspective in women’s health; social and economic factors serve as both causes and consequences of these co-conditions.
- The recognition of social forces as part of diabetes and depression aetiology in low- and middle-income countries presents new challenges for public health because it underscores that medicating these complex illnesses does not fully address them; this requires that we understand the limitations, and harness the power of, cultural beliefs and social conditions to shape behaviors that affect chronic diseases.

**Key messages for action**

- Integrating a social and psychological approach into health agendas, from the clinical to the policy level, can make a big impact.
- Strategic points of intervention can improve women’s social and emotional well being across the life course, which could then empower them to identify and care for their own health problems more effectively.
- With relatively little additional investment, basic mental healthcare (as illustrated in the WHO mental health Gap Action Program (mhGAP)) can be integrated into existing diabetes care guidelines; such an approach is particularly important for women who face a higher burden of social problems and mental illness, which influence diabetes self-care and health outcomes.
